# Marked increase in etravirine and saquinavir plasma concentrations during atovaquone/proguanil prophylaxis

**DOI:** 10.1186/1475-2875-10-141

**Published:** 2011-05-21

**Authors:** Chiara Tommasi, Rita Bellagamba, Massimo Tempestilli, Antonio D'Avolio, Anna L Gallo, Jelena Ivanovic, Emanuele Nicastri, Leopoldo P Pucillo, Pasquale Narciso

**Affiliations:** 1 "National Institute for Infectious Diseases "L. Spallanzani", Via Portuense 292, 00149 Rome, Italy; 2 Clinica Universitaria c/o Ospedale Amedeo di Savoia, University of Turin, Turin - Italy

## Abstract

The case of a 32-year-old Caucasian female with multi-drug resistant HIV-1 subtype B infection treated with a salvage regimen including maraviroc, raltegravir, etravirine and unboosted saquinavir who started atovaquone/proguanil prophylaxis, is reported. The potential interactions between atovaquone/proguanil and these anti-retroviral drugs are investigated. Pharmacokinetic analyses documented a marked increase in etravirine and saquinavir plasma concentrations (+55% and +274%, respectively), but not in raltegravir and maraviroc plasma concentrations. The evidence that atovaquone/proguanil significantly interacts with etravirine and saquinavir, but not with raltegravir and maraviroc, suggests that the mechanism of interaction is related to cytochrome P450.

## Background

Atovaquone/proguanil (Malarone^®^) is a fixed-dose combination of the anti-malarial agents atovaquone and proguanil hydrochloride. HIV-infected travellers in malaria endemic countries frequently use atovaquone/proguanil as a prophylaxis.

Atovaquone displays linear pharmacokinetic with a mean absolute bioavailability of 23%. It is highly protein-bound (> 99%) but does not displace other highly protein-bound drugs in vitro [1]. The principal excretion route is the liver, with the 94% of the drug excreted unchanged in the faeces. The elimination half-life is 2-3 days in adults [1].

Proguanil is rapidly absorbed from the gastrointestinal tract and achieves peak plasma concentrations in 2-4 hours, with an absolute bioavailability as high as 60% [1]. It is 75% protein bound, and this binding is unaffected by the presence of atovaquone and *vice versa *[1]. Proguanil is metabolized to cycloguanil (primarily trough CYP2C19) and 4-chlorophenylbiguanide, with between 40% and 60% of proguanil excreted renally. The elimination half-life of proguanil is 12-21 hours [1].

Drug interactions between atovaquone/proguanil and tetracycline, metoclopramide, rifampin, rifabutin and warfarin have been described. The concomitant administration of indinavir is associated with a 23% decrease in indinavir C_min _(90% CI 8-35%). Potential interactions between proguanil and other drugs that are CYP2C19 substrates or inhibitors are unknown [1].

## Case presentation

A 32-year-old Caucasian female was admitted to the "L. Spallanzani" National Institute for Infectious Diseases in Rome for multi-drug resistant HIV-1 subtype B infection (diagnosed in 1985), beta-thalassaemia, severe pulmonary hypertension, wasting syndrome and ritonavir allergy. In October 2007, a genotypic resistance test (GRT) revealed high-level resistance to all currently available anti-retrovirals (ARVs) and a salvage treatment with unboosted darunavir (600 mg bid), lamivudine (300 mg qd) and raltegravir (400 mg bid) was started. Three months later, a viral rebound occurred; a new GRT evidenced the emergence of primary N155H/N and secondary D232N mutations in integrase gene with no new RT and PR-related mutations. In March 2008, after the viral tropism assay, a new ARV regimen including raltegravir (400 mg bid), saquinavir (1000 mg bid), maraviroc (150 mg bid) and etravirine (200 mg bid) was started. Viremia immediately decreased to below the detection limit (50cp/ml) and remained undetectable. Tolerability was good and no grade 3/4 adverse events have been reported.

**Figure 1:**
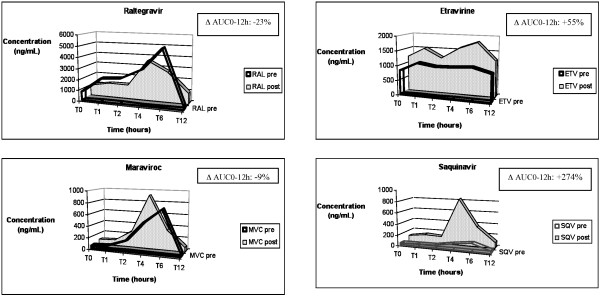
**Plasma concentrations of antiretroviral drugs (raltegravir, maraviroc, etravirine and saquinavir) evaluated as Area Under Curve (AUC) before starting atovaquone/proguanil (pre) and at day 20 of prophylaxis (post)**. The D AUC0-12h represents the difference in AUC0-12h (during prophylaxis AUC minus pre-prophylaxis AUC). An HPLC-UV method for the simultaneous quantification of the HIV integrase inhibitor raltegravir, the new non- nucleoside reverse transcriptase inhibitor etravirine, protease inhibitor saquinavir and CCR-5 inhibitor maraviroc was used [11,12].

In September 2009, the patient planned to spend a two-week holiday in Kenya. The CD4 cell count was 334/mmc. Standard malaria prophylaxis with atovaquone/proguanil fixed-dose combination (250/100 mg) was prescribed: one tablet daily starting two days before the journey and ending the treatment seven days after return to Italy [2]. A 12 hour-PK of anti-retrovirals was performed before starting prophylaxis with atovaquone/proguanil (Figure 1). The samples were collected in fasting status following this order: the first sample 12 hours after the last dose of anti-retrovirals (pre-dose); the second sample one hour after breakfast and anti-retrovirals consumption; the following samples at 2, 4, 6 and 12 hours post anti-retrovirals' intake. The second PK for the determination of anti-retrovirals' plasma concentrations during atovaquone/proguanil prophylaxis was collected at day 20 on prophylaxis with the same timing plan (Figure 1). In order to avoid any food interference with the PK results, the same diet was administered in both the PK sampling days. During the stay in Kenya, the adherence was monitored by self-reported diary that documented an adherence level of 100% both for anti-retroviral and prophylaxis drugs. This data was retrospectively confirmed by pills count system.

Twenty days after atovaquone/proguanil was started, the blood examinations documented a CD4 count of 438 cell/mmc, with undetectable HIV-RNA. Liver and haematological parameters were normal. No clinical or laboratory adverse events were reported during or after the journey.

## Conclusions

In a recent study, Van Luin *et al *demonstrated that geometric mean ratios (95% confidence interval) of the area under the curve (AUC) of atovaquone in patients on efavirenz, lopinavir/ritonavir and atazanavir/ritonavir compared with healthy volunteers were 0.25 (0.16-0.38), 0.26 (0.17-0.41) and 0.54 (0.35-0.83), respectively. Proguanil plasma concentrations were also significantly lower (38-43%). No data on anti-retroviral pharmacokinetics during atovaquone/proguanil prophylaxis were provided. The authors concluded that physicians should be alert to atovaquone/proguanil prophylaxis failures in patients taking efavirenz, lopinavir/ritonavir or atazanavir/ritonavir [3]. The anti-retrovirals' pharmacokinetic during atovaquone/proguanil prophylaxis were analysed. A marked increase in etravirine and saquinavir AUC0-12h (+55% and +274%, respectively) and a slight decrease in raltegravir and maraviroc AUC0-12h (-23% and -9%, respectively) were documented. Although AUCs between pre- and post-prophylaxis are not very different, in the PK profiles of maraviroc and raltegravir the Tmax and the Cmax in post- prophylaxis appear to be earlier than in pre- prophylaxis, suggesting a more fast absorption of both drugs. Unfortunately, it was not possible to determine atovaquone and proguanil plasma concentrations. However, the absence of established minimum effective atovaquone plasma concentrations in the setting of malaria prophylaxis makes it difficult to assess the clinical relevance of dosing atovaquone and proguanil plasma concentrations, except for the evaluation of potential interactions with anti-retroviral drugs [3]. The evidence that atovaquone/proguanil significantly interacts with etravirine and saquinavir, but not with raltegravir and maraviroc, suggests that the mechanism of interaction is related to cytochrome P450. Etravirine is metabolized primarily by hydroxylation through several cytochrome P-450 isoenzymes (mainly CYP3A, CYP2C9 and CYP2C19), followed by glucuronidation of the metabolites. It is also an inducer of CYP3A and a mild inhibitor of CYP2C9, 2C19 and P-glycoprotein [4]. Saquinavir is metabolized by CYP3A4. It is neither an inducer nor an inhibitor of P450 system, but it is substrate and inhibitor of P-glycoprotein and MRP1 [5,6]. Raltegravir is metabolized via UGT1A1-mediated glucuronidation and is neither an inducer nor an inhibitor of the CYP oxidation system or P-glycoprotein [7]. Maraviroc is extensively metabolized by CYP3A4 and it is a substrate for P-glycoprotein. At higher doses, maraviroc is a potential inhibitor of CYP2D6 and perhaps of P-glycoprotein [8]. In vitro, maraviroc was not found to inhibit any major CYP isoenzyme, including CYPIA2, CYP2B6, CYP2C8, CYP2C19, CYP2D6, and CYP3A4 [9]. Basing on the pharmacokinetic characteristics of these anti-retrovirals, it is supposable that atovaquone, or more likely proguanil, interferes with etravirine and saquinavir for CYP450 mediated metabolism (perhaps on the 2C isoenzymes) with a consequent marked increase in plasma concentrations of both anti-retrovirals. Despite of the clear effect in plasma concentrations of etravirine and saquinavir, in this case the patient noticed no side effects, and blood examinations during prophylaxis documented any lab toxicities. It is possible that, in this case, the absence of drug-related toxicity was due to the use of unboosted protease inhibitor; while for etravirine there are no clear data on the correlation between PK and toxicity [10].

This case should alert the physicians to be cautious and vigilant when atovaquone/proguanil prophylaxis is prescribed in patients treated with etravirine and/or saquinavir (especially if ritonavir-boosted).

## Competing interests

The authors declare that they have no competing interests.

## Authors' contributions

CT conceived of the study, performed blood sampling and drafted the manuscript. RB, JI and EN were responsible for the clinical management of the patient. MT, ALG and AD carried out the pharmacokinetic studies. LP and PN participated in study design and coordination. All authors read and approved the final manuscript.
